# Quantum dot-induced cell death involves Fas upregulation and lipid peroxidation in human neuroblastoma cells

**DOI:** 10.1186/1477-3155-5-1

**Published:** 2007-02-12

**Authors:** Angela O Choi, Sung Ju Cho, Julie Desbarats, Jasmina Lovrić, Dusica Maysinger

**Affiliations:** 1 Department of Pharmacology & Therapeutics, McGill University, 3655 Promenade Sir William-Osler, McIntyre Medical Sciences Building, Montreal, QC, H3G 1Y6, Canada; 2 Faculty of Pharmacy and Department of Chemistry, University of Montreal, Pavillon J. A. Bombardier, C.P. 6128 Succursale Centre-Ville, Montreal, QC, H3C 3J7, Canada; 3 Department of Physiology, McGill University, Montreal, QC, H3G 1Y6, Canada

## Abstract

**Background:**

Neuroblastoma, a frequently occurring solid tumour in children, remains a therapeutic challenge as existing imaging tools are inadequate for proper and accurate diagnosis, resulting in treatment failures. Nanoparticles have recently been introduced to the field of cancer research and promise remarkable improvements in diagnostics, targeting and drug delivery. Among these nanoparticles, quantum dots (QDs) are highly appealing due to their manipulatable surfaces, yielding multifunctional QDs applicable in different biological models. The biocompatibility of these QDs, however, remains questionable.

**Results:**

We show here that QD surface modifications with N-acetylcysteine (NAC) alter QD physical and biological properties. In human neuroblastoma (SH-SY5Y) cells, NAC modified QDs were internalized to a lesser extent and were less cytotoxic than unmodified QDs. Cytotoxicity was correlated with Fas upregulation on the surface of treated cells. Alongside the increased expression of Fas, QD treated cells had increased membrane lipid peroxidation, as measured by the fluorescent BODIPY-C_11 _dye. Moreover, peroxidized lipids were detected at the mitochondrial level, contributing to the impairment of mitochondrial functions as shown by the MTT reduction assay and imaged with confocal microscopy using the fluorescent JC-1 dye.

**Conclusion:**

QD core and surface compositions, as well as QD stability, all influence nanoparticle internalization and the consequent cytotoxicity. Cadmium telluride QD-induced toxicity involves the upregulation of the Fas receptor and lipid peroxidation, leading to impaired neuroblastoma cell functions. Further improvements of nanoparticles and our understanding of the underlying mechanisms of QD-toxicity are critical for the development of new nanotherapeutics or diagnostics in nano-oncology.

## Background

Neuroblastoma is the most frequently occurring extracranial solid tumour in children, accounting for 9% of all childhood cancers, with poor prognosis [1]. This malignant tumour arises from neuroepithelial cells of the sympathetic nervous system early in development, and is typically found in the adrenal medulla, abdomen, chest or neck [2]. Neuroblastoma, however, remains a therapeutic challenge as current surgical and chemical treatments are insufficient to prevent tumour recurrence, metastasis and progression [3]. Accurate disease staging is critical for appropriate therapeutic intervention, but existing imaging tools are still lacking in early and accurate diagnosis [4].

The introduction of nanoparticles in the field of cancer research has recently improved diagnosis, targeting and drug delivery with the use of nanotubes, liposomes, dendrimers and polymers [5-7]. Other nanoparticles, such as quantum dots, possess excellent photophysical properties and prove to be an elegant alternative to the traditional bioimaging tools [8]. Quantum dots (QDs) are one of the most rapidly evolving products of nanotechnology, with great potential as a tool for biomedical and bioanalytical imaging. Their superior photophysical properties [9] and sometimes multifunctional surfaces are suitable for applications in various biological models [10]. A study by the Nie group describes the application of these multifunctional QDs for *in vivo *imaging and targeting of breast and prostate cancers [11]. Although the development of QDs as bioimaging tools may be well underway, their potential application as therapeutic agents is yet to be explored. Biological media, intracellular microenvironment and different enzymatic systems could destabilize originally well protected QD surfaces yielding more cytotoxic nanoparticles [12,13]. Uncoated or weakly stabilized cadmium telluride QDs produce significant amounts of reactive oxygen species *in vitro *[12], and induce death in various cell types [14,15].

Oxidative stress-induced cell death, both apoptosis and necrosis, can involve a number of cellular mechanisms, one of which includes the activation of Fas receptor [16,17]. Fas (CD95) belongs to the family of tumour necrosis factor receptors, and is a prototypical "death receptor." In the immune system, it regulates cell numbers by inducing apoptosis, and is involved in T cell-mediated cytotoxicity. It can also induce neuronal cell death [18-21]. Activation of Fas receptor by Fas ligand recruits the Fas-Associated Death Domain (FADD) to the Death Domain in the cytoplasmic tail of the receptor, and can lead to caspase activation and cell death [22]. Downstream signaling of Fas can also induce activation of lipases and pro-apoptotic transcription factors like p53, which then potentiate apoptosis [23].

Oxidative stress can also induce other levels of cell membrane damage, including membrane lipid peroxidation [24]. Free radicals induce the cleavage of membrane lipids, resulting in the production of aldehydes, reinforcing cellular stress. Intracellular lipid peroxidation can also occur at the level of the organelle membranes, especially at the membranes of the highly metabolically active mitochondria. Mitochondria regulate crucial cellular processes including adenosine triphosphate (ATP) production, intracellular pH regulation and neuronal-glial interactions [25]. Many neurodegenerative diseases, including Parkinson's and Alzheimer's diseases, involve the malfunctioning of the mitochondria, seen as decreased mitochondrial activity, decreased ATP production or loss of mitochondrial membrane potential (Δψ_m_) [25].

In this study, we explored mechanisms of QD-induced toxicity in a human neuroblastoma cell line exposed to cysteamine-QDs and QDs modified by an antioxidant, N-acetylcysteine. We report new mechanisms of cytotoxicity induced by these QDs, including the i) upregulation of the Fas receptor, ii) lipid peroxidation, and iii) impaired mitochondrial function. Understanding the mechanisms underlying QD-toxicity will provide alternative ways of nanoparticle manipulations to make them more suitable tools in nanomedicine, specifically nano-oncology.

## Results

### Surface modifications of cadmium telluride QDs with N-acetylcysteine

**Figure 1:**
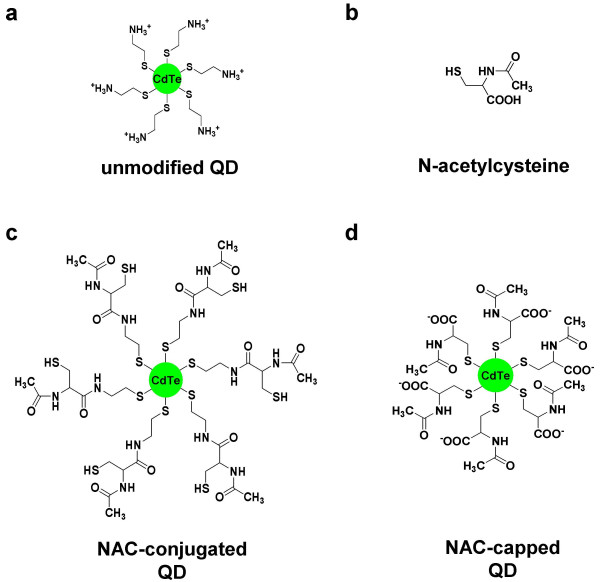
**Schematic representations of unmodified and NAC-modified QDs**. **a. **cysteamine-capped ("unmodified") QD (λ_em _= 542 nm in water), **b**. N-acetylcysteine (NAC) **c**. NAC-conjugated QD (λ_em _= 526 nm in water), **d**. NAC-capped QD (λ_em _= 528 nm in water).

To investigate mechanisms underlying cell death induced by cadmium telluride (CdTe) QDs, we modified the surface of cysteamine-capped CdTe QDs with an antioxidant, N-acetylcysteine (NAC, Figure 1b), a drug which has been found previously to protect cells against oxidative stress and QD-induced cytotoxicity [14]. Cysteamine-capped (''unmodified'') QDs (Figure 1a) have amino groups at the surface and are positively charged (+14.2 mV). Covalent binding of NAC to cysteamine on the QD surface (Figure 1c) yielded NAC-conjugated QDs with a decreased net surface charge, and charge-charge complexation of NAC and cysteamine yielded NAC-capped QDs with carboxylic groups on the surface and a net negative charge of -9.8 mV (Figure 1d). Spectrofluorometric measurements revealed marked differences in the fluorescence intensities and QD stability in different media (see Additional file 1). In phosphate buffered saline (PBS), cysteamine-QDs show a red-shift with time but no change in fluorescence intensity, whereas, NAC-conjugated QDs decreased in fluorescence with time. NAC-capped QDs were the most stable in PBS, with no spectral shift and no loss of fluorescence within 24 hours.

### Cytotoxicity of NAC-modified CdTe QDs in neuroblastomas

**Figure 2:**
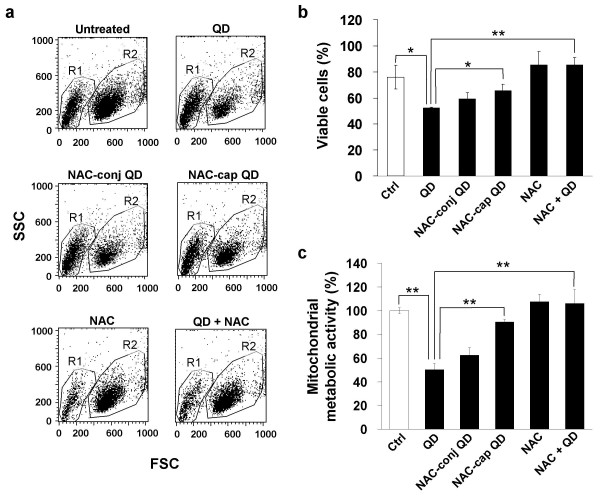
**Viability and metabolic activity of human neuroblastoma (SH-SY5Y) cells treated with NAC-modified and unmodified cadmium telluride QDs**. **a**. Quantum dot toxicity differs depending on their surface modifications by NAC. Flow cytometry light scatter dot plots reveal two distinct cell populations corresponding to viable cells (R1), and cells in various stages of apoptotic death (R2). FSC, forward scatter (proportional to cell size); SSC, side scatter (proportional to cell complexity or granularity). **b**. Cell death in neuroblastomas after 24 hours of QD treatments. Graph shows percentage of dead cells (gated on R2) for each treatment: Ctrl = cells under serum-deprivation with no drug or QD added; QD = cysteamine-QDs; NAC-conj QD = NAC-conjugated QDs; NAC-cap QD = NAC-capped QDs; NAC (2 mM); NAC + QD (2 mM NAC + 5 μg/mL cysteamine-QD). All QDs were added at 5 μg/mL. Mean values and standard deviations from three independent experiments (N = 9) are shown. (*p < 0.05; **p < 0.01). **c**. Mitochondrial metabolic activity was assessed using MTT and its conversion to formazan was measured at 595 nm. All values are expressed relative to cells without any drug or QD addition (Ctrl) taken as 100%. Note significant decrease with QD treatments and full recovery in the presence of 2 mM NAC. Mean values and standard deviations from quadruplicate measurements in two independent experiments (N = 8) are shown. (**p < 0.01).

To examine the cytotoxicity of cysteamine-QDs and NAC-modified QDs, we assessed the viability of SH-SY5Y human neuroblastoma cells by fluorescence-activated cell scanning (FACS), and their mitochondrial metabolism using a MTT reduction assay. Our FACS data show that cells exposed to 5 μg/mL of cysteamine-QDs, NAC-conjugated or NAC-capped QDs yielded distinct populations of dead cells (Figure 2a), suggesting significant toxicity induced by these QDs. Significantly less viability was observed in cells treated with cysteamine-QDs (52.2 ± 0.7%, *p *< 0.05) when compared to untreated control cells in serum-free medium (75.9 ± 9.1%). It is noteworthy that trophic factor deprivation, due to serum withdrawal, contributes to cell death which explains the approximately 25% decrease in viability in the absence of QDs (i.e. untreated control). NAC treatment can rescue cells in this trophic factor withdrawal paradigm [26]. QD-induced cytotoxicity is prevented with cell pretreatment with 2 mM NAC (85.5 ± 5.7%; *p *< 0.01; Figure 2b), confirming and complementing results from our previous studies demonstrating the effectiveness of NAC against both trophic factor deprivation and additional QD-insult [14]. These multiple insults to neuroblastoma cells lead to cell death both by apoptosis and necrosis. The latter is characterized by mitochondrial and lysosomal swelling and perinuclear localization of these organelles [17]. NAC-capped and NAC-conjugated QDs are still cytotoxic (65.6 ± 5.0%, *p *< 0.05 and 59.1 ± 5.1%, *p *< 0.05 respectively) compared to control.

Results of the FACS analyses were corroborated by data from measuring cellular MTT reduction (Figure 2c). Mitochondrial metabolic activity was most significantly reduced in cells in the presence of cysteamine-QDs (50.1 ± 5.2%; *p *< 0.01). NAC-conjugated QDs also significantly reduced the cellular mitochondrial activity (62.3 ± 6.5%; *p *< 0.01) compared to control. Cells treated with NAC-capped QDs, on the other hand, suffered less cytotoxic damage and showed significantly higher mitochondrial metabolic activity (90.2 ± 2.2%; p < 0.01) compared to cells treated with cysteamine-QDs or NAC-conjugated QDs. Cells pretreated with NAC, prior to cysteamine-QD addition, show significantly higher activity (106.1 ± 11.8%; *p *< 0.01) when compared to QD-treated cells, again reinforcing the protective role of free NAC against QD-induced toxicity.

### Upregulation of Fas at the cell surface and internalization of QDs by neuroblastoma cells

**Figure 3:**
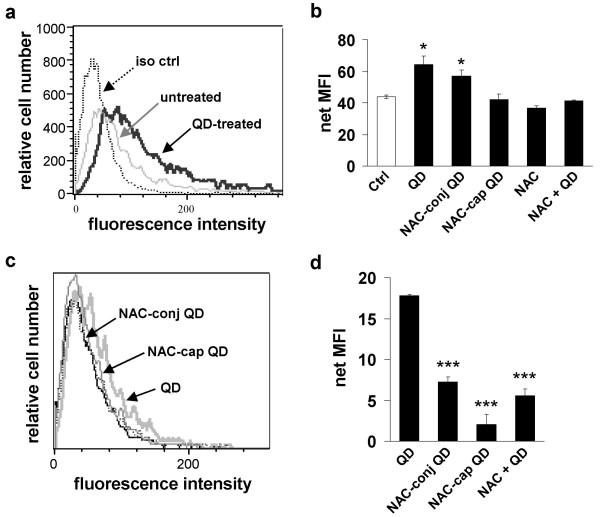
**Fas expression and internalization of QDs**. **a**. Exposure to QDs induces cell-surface Fas expression in neuroblastomas. Fas expression was assessed by FACS in untreated cells (grey line) and in cells exposed to cysteamine-QDs for 24 hours (black line). Dotted line shows background staining of untreated cells with isotype-matched control antibody. **b**. Net Fas expression was calculated as Mean Fluorescence Intensity (MFI) of cells stained with anti-Fas antibodies subtracted by MFI of isotype control antibody-stained cells. Averages and standard deviations from three independent experiments (N = 9) are shown (*p < 0.05). **c**. QD uptake was assessed by flow cytometry in neuroblastoma cells treated with 5 μg/ml QDs (unmodified and NAC-modified) for 24 hours. **d**. Net Mean Fluorescence Intensities (MFI) of cells treated with cysteamine-QDs, NAC-conjugated and NAC-capped QDs, and cysteamine-QDs in the presence of 2 mM NAC (NAC + QD) are shown. Net MFI was calculated as MFI of QD-treated cells subtracted by the autofluorescence of untreated cells. Note a significant decrease (p < 0.05) in the MFI of NAC-modified QDs compared with MFI of cysteamine-QDs. Averages and standard deviations from three independent experiments (N = 9) are shown (***p < 0.001).

QD-induced cytotoxicity involves oxidative stress, specifically via the production of reactive oxygen species (ROS) [12,15]. One cell-damaging, downstream effect of ROS production is the upregulation of the cell surface Fas receptor. FACS analyses revealed significant upregulation of Fas expression on the surface of SH-SY5Y cells treated with cysteamine-QDs (net mean fluorescence intensity (MFI) is 64.3 ± 5.5, *p *< 0.05) and NAC-conjugated QDs (net MFI = 57.1 ± 3.7, *p *< 0.05) when compared to untreated control cells (net MFI = 43.9 ± 1.1; Figures 3a and 3b). No upregulation of Fas was observed in cells treated with NAC-capped QDs (net MFI = 42.1 ± 3.7), and Fas upregulation was completely inhibited in cells pretreated with NAC in the presence of cysteamine-QDs (net MFI = 41.5 ± 0.4), suggesting that QD-induced Fas expression is likely due to QD-mediated oxidative stress.

In addition, free Cd^2+ ^released from QDs and the extent of QD uptake can contribute to the cell damage and eventually cell death. We measured intracellular and extracellular Cd^2+ ^concentrations in SH-SY5Y cell cultures treated with QDs. Results from this study and from our recently published study [27] show that Cd^2+ ^concentrations contribute to, but cannot fully explain QD-induced cytotoxicity (31.1 ± 1.7% and 58.0 ± 2.1% cytotoxicity induced by Cd^2+ ^and QDs respectively), suggesting that impairment of cellular functions by QDs is multifactorial.

The extent of QD uptake was assessed by FACS analyses. Cells treated with cysteamine-QDs, NAC-conjugated and NAC-capped QDs show marked differences in QD uptake. In particular, cysteamine-QD-treated cells show an evident shift in fluorescence intensity compared to the untreated control and to both NAC-conjugated and NAC-capped QD-treated cells (Figure 3c). Quantitative measurements of the mean fluorescence intensity show that cysteamine-QDs were indeed taken up most avidly (net MFI = 17.8 ± 0.1; Figure 3d). On the other hand, NAC-conjugated QDs (net MFI = 7.3 ± 0.6; *p *< 0.001) and NAC-capped QDs (net MFI = 2.1 ± 1.2; *p *< 0.001) were internalized significantly less than cysteamine-QDs. The net MFI for cells pretreated with 2 mM NAC (5.6 ± 0.8; *p *< 0.001) was significantly lower than in the absence of NAC (net MFI = 17.8 ± 0.1), suggesting that NAC either reduced QD uptake or partly quenched QD fluorescence. The latter is unlikely as spectral data show that these NAC-modified QDs have comparable, and in some cases even higher, fluorescence intensities as the unmodified QDs (see Additional file 1). On the other hand, measurements of intracellular Cd^2+ ^show reduced Cd^2+ ^content in NAC pretreated cells, supporting the notion that less QDs were internalized by the cells and that extracellular Cd^2+ ^effects were also diminished by NAC.

### QD-induced lipid peroxidation and change in membrane potential (Δψ_m_) of the mitochondria

**Figure 4:**
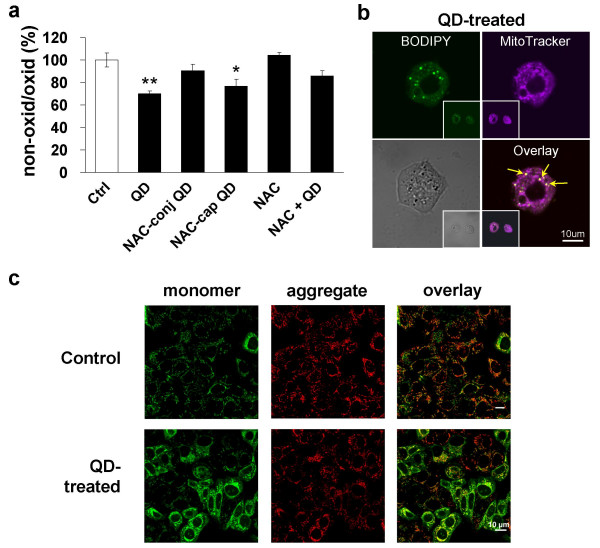
**QD-induced mitochondrial lipid peroxidation and change in membrane potential**. **a**. Spectrofluorometric assessment of lipid membrane peroxidation by ratiometric approach in untreated (Ctrl) or QD-treated cells. The ratio between the red and green fluorescence in the control was taken as 100% and all other values with NAC or QD treatments were expressed relative to it. All values are means from quadruplicate measurements and are obtained from three independent (N = 12) experiments (*p < 0.05; **p < 0.01). **b**. Confocal micrograph showing dual labeling of oxidized lipids (green fluorescence from oxidized BODIPY-C_11_) within mitochondria (labeled with MitoTracker Deep Red 633). Insets show two adjacent cells from the same field. Scale bar = 10 μm. **c. **Confocal micrographs of SH-SY5Y cells labeled with JC-1 reveal decrease in mitochondrial membrane potential after QD treatment. Cells were treated with 5 μg/mL QD and typical change in fluorescence from red (Em = 590 nm) to green (Em = 530 nm) was assessed in cell cultures in serum-free medium (control) or QD (5 μg/mL). Note an enhanced intensity of green fluorescence in QD-treated cells. The micrograph illustrates the loss in mitochondrial potential upon oxidative stress induced by QDs. Scale bar = 10 μm.

The subcellular distribution of internalized QDs has previously been reported to induce ROS production and organelle damage [12,14]. Here we identify two intracellular targets of this QD-induced ROS, namely membrane lipids and mitochondria. In response to oxidative stress, cell surface and organellar membrane lipids may undergo peroxidation [24]. We assessed lipid peroxidation by spectrofluorometric measurements of the fluorescent BODIPY-C_11 _dye and by confocal microscopy (Figures 4a, b). When compared to untreated control cells (100.0 ± 6.3%), cells treated either with cysteamine-QDs (70.2 ± 2.0%, *p *< 0.01) or NAC-capped QDs (76.6 ± 6.5%, *p *< 0.05) showed significantly reduced red (non-oxidized) to green (oxidized) ratios. Cells treated with NAC-conjugated QDs or pretreated with free NAC, in the presence of cysteamine-QDs, did not show significant lipid peroxidation compared to the untreated control.

Double labeling using BODIPY-C_11 _dye and MitoTracker Deep Red 633 revealed lipid peroxidation of the mitochondrial membranes as shown by confocal microscopy (Figure 4b). Co-localized oxidized BODIPY-C_11_(green) and MitoTracker Deep Red 633 (red-purple) appear as punctate yellow signals, suggesting local membrane lipid peroxidation within the mitochondria in cells treated with QDs.

Membrane lipid peroxidation can produce damaging aldehydes, and at the mitochondrial level, this can impair mitochondrial functions [28]. Confocal microscopy analyses of cells stained with JC-1 clearly show that QD-treated cells have significantly reduced mitochondrial membrane potential (Δψ_m_) (Figure 4c). Compared to the strong red fluorescence of JC-1 aggregates observed in the untreated control, QD-treated cells show an increased intensity in green fluorescence (JC-1 monomers) which correlates with a decrease in Δψ_m_.

## Discussion

Initial reports on the potential toxicity of some types of quantum dots (QDs) [13,14,29] prompted the development of differently modified QDs as tools in the biological sciences. Several studies describing modifications to improve QD biocompatibility for their broad applications in the medical sciences were recently reported [10,30,31]. At the other end of the spectrum, research groups are also attempting to harness and apply QD toxicity in toxicotherapy. For instance, one study proposed the application of dopamine-conjugated QDs as inducers of cellular phototoxicity [32], while others are using QDs in photodynamic therapy [33] and to target different stages of cancers [11,34]. Using NAC-conjugated, NAC-capped and cysteamine-capped CdTe QDs, this study shows several cellular responses of human neuroblastoma cells to these nanoparticles. Surface-modified nanoparticles with NAC led to reduced cell death, decreased Fas expression and decreased mitochondrial membrane lipid peroxidation. The negatively charged NAC-capped QDs were the most benign, followed by NAC-conjugated QDs. Cysteamine-QDs, with a net positive surface charge, showed significant cellular uptake, as well as increased upregulation of Fas receptors on the cell surface and membrane lipid peroxidation, contributing to the impairment of mitochondrial and overall cell functions. In addition to surface charge, cytotoxicity is also affected by other physicochemical properties, including particle size, core-shell composition and capping [14,35,36].

QD biocompatibility can be easily altered by surface modifications, such as conjugation and capping with biomolecules and polymers [31,37,38]. The Hoshino group characterized the physicochemical properties of different surface-modified CdSe QDs and reported that these surface modifications affect QD surface potential, QD fluorescence and QD-induced cytotoxicity [36]. In our study, we found that QD surface conjugation and capping with an antioxidant, N-acetylcysteine (NAC), reduced QD uptake and cytotoxicity (Figures 2 and 3). Moreover, pretreatment of cells with free NAC fully protected cells from QD-induced cytotoxicity (Figure 2b), as demonstrated in our previous study in a different cell line [12]. NAC can protect cells both from apoptosis and necrosis. Mechanisms of the cytoprotective action of NAC are well-documented, and involve NAC acting (i) as a direct thiol antioxidant, (ii) as a glutathione precursor, (iii) as a transcription regulator for genes involved in cellular homeostasis, and (iv) as a cell survival promoter via inhibition of apoptotic pathways including JNK and p38 [26].

Highly metabolically active mitochondria are particularly sensitive and vulnerable targets to cellular stress [25]. Cells treated with QDs undergo a change in mitochondrial membrane potential (Δψ_m_) (Figure 4c). Membrane depolarization has been widely associated with the release of the apoptotic factor, cytochrome c, which amplifies pro-apoptotic caspase cascades, promoting cell death [12,25]. Among the regulators of mitochondrial membrane potential, cardiolipin, a mitochondrial membrane specific lipoprotein, is of particular relevance in neuronal cells [39,40]. The abundance of cardiolipin in the membranes of the mitochondria maintains the membrane potential and regulates the release of cytochrome c. Upon cellular stress, cardiolipin, along with other membrane lipids, is degraded due to lipid peroxidation, and the membrane potential is no longer stable, resulting in an uncontrolled release of mitochondrial content [40,41]. In addition to causing membrane instability and increasing the vulnerability of the cell to subsequent insults [28], lipid peroxidation can also generate harmful and relatively stable aldehyde products which add to the oxidative stress. One of these damaging aldehydes is 4-oxo-2-nonenal (ONE), which acts by activation of the p53 signaling pathway and induces apoptosis in SH-SY5Y neuroblastoma cells [42].

Besides intracellular targeting at the mitochondrial level, QD treatment leads to an upregulation of cell surface Fas expression (Figures 3a and 3b). The Fas receptor, when activated by Fas ligand, associates with FADD which recruits caspase-8 or caspase-10, and forms the death-inducing signaling complex (DISC). Caspases-8/10 autocatalyze their own cleavage [43-45], triggering a cascade of caspase activation that culminates in apoptosis. This cascade may be further amplified by cleavage of the caspase-8/10 substrate Bid, which then inserts into the mitochondrial membrane, resulting in loss of Δψ_m _and release of cytochrome c, further accelerating apoptosis. Fas has been implicated as an inducer of apoptosis under conditions of high *in vivo *oxidative stress [46-48], and recent studies show that Fas expression may also be triggered upon activation of proapoptotic transcription factors, such as FOXO3 [49].

Nanoparticles, such as the CdTe QDs investigated here, enter cells and can get sequestered within different organelles, changing organellar morphologies and obstructing their functions, leading eventually to cell death of different types [17,50,51]. For instance, our recent study in human breast cancer cells [27] showed that QDs induce enlargements of lysosomes and mitochondria, both of which are morphological indications of necrotic cell death. On the other hand, intracellular accumulation of unprotected or unstable QDs can eventually result in QD degradation and Cd^2+ ^release from the QD core, initiating apoptosis. The extent of apoptosis in neuroblastoma cells and Cd^2+ ^released are, however, not strongly correlated, suggesting additional contributors to cell death aside from the free Cd^2+^.

Neuroblastoma cells that were deprived of serum-derived trophic factors are more susceptible to additional insults induced by QDs (i.e. ROS, Cd^2+^), leading to both type I (apoptosis) and type III (necrosis) cell death [17]. Under the circumstances in which QDs could be employed for the detection and elimination of neuroblastoma, one should bear in mind not only the physical properties of QDs but also the vulnerability of healthy tissues surrounding the tumour, the rate of QD sequestration and the rate of metal elimination from the body [51]. Collectively, earlier and current findings suggest that cell pre-conditioning, combined with modifications of the QD surface with NAC and a tumour-specific ligand (e.g. Trk mimetics to target Trk receptors) could yield an improved nano-oncological therapeutic, sensitizing or diagnostic agent for neuroblastomas.

## Conclusion

**Figure 5:**
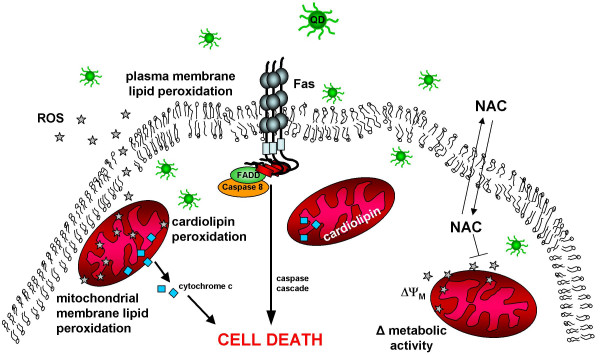
**Proposed mechanism of QD induced cell death involving Fas, lipid peroxidation and mitochondrial impairment**. Cells exposed to cadmium telluride quantum dots (unmodified and NAC-modified) induce ROS which causes Fas upregulation and plasma membrane lipid peroxidation. Apoptotic cell death is induced by activation of Fas and its downstream effectors. Lipid peroxidation also occurs at the mitochondrial membranes, degrading cardiolipin, changing the mitochondrial membrane potential, eventually leading to the release of cytochrome c [12], and promoting apoptotic cascades. NAC bound to the QD surface, modifies the extent of QD internalization, which is correlated with cell death, upregulation of Fas, and ROS induced lipid peroxidation. NAC treatment (2–5 mM) abolishes oxidative stress, induces antioxidant enzymes and attenuates mitochondrial impairment.

Results from this study provide new mechanistic data (summarized in Figure 5) on the much debated issue of QD toxicity. Cadmium telluride (CdTe) QD-induced cytotoxicity depends on multiple QD properties including QD core size, stability in biological media and surface chemistry which determine the extent of cellular internalization. Mechanisms of CdTe QD-induced toxicity include multiple organelle damage and involve increased Fas receptor expression and cell membrane lipid peroxidation in SH-SY5Y neuroblastoma cells. These damages bring about cell death both by apoptosis and necrosis. Understanding the mechanisms underlying QD toxicity is important as QDs and other nanoparticles are promising tools in the field of nano-oncology as potential imaging agents, photosensitizers, biosensors and nanotherapeutics.

## Materials and methods

### Preparation of CdTe quantum dots

Tellurium powder (200 mesh, 99.8%), sodium borohydride (99%), cadmium perchlorate hydrate, N-acetylcysteine (99%) and cysteamine hydrochloride (98%) were purchased from Sigma-Aldrich. Milli-Q water (Millipore) was used as a solvent. Photoluminescence measurements were carried out at room temperature using a Cary Eclipse Fluorescence spectrometer. The excitation wavelength was set at 400 nm. The excitation and emission slits were set at 5 nm. Dialysis was performed using spectra/por molecularporous membrane tubing (Spectrum Laboratories, Inc.) with a 6000–8000 Da molecular weight cutoff. Centrifugation was performed with Eppendorf centrifuge 5403 (10,000 rpm) and Eppendorf centrifuge 5415 C (14,000 rpm).

### Preparation of Cysteamine capped (+) CdTe

Sodium borohydride (0.8 g, 21.1 mmol) was dissolved in water (20 mL) at 0°C under N_2 _atmosphere. Tellurium powder (1.28 g, 10 mmol) was added portionwise and the mixture was stirred at 0°C for 8 h under N_2 _atmosphere. The reaction mixture was stored at 4°C in the dark and used in the next step.

The thiol capped-QDs were prepared as described [12]. Briefly, cadmium perchlorate hydrate (500 μL, 1 M aqueous solution) and cysteamine hydrochloride (300 mg, 2.64 mmol) were dissolved in 200 mL of N_2 _saturated Milli-Q water. The pH of the solution was adjusted to 5.1 with 1N NaOH aqueous solution prior to addition of an aliquot of the previously prepared NaHTe solution (200 μL). The reaction mixture was heated to reflux for 25 min under N_2_. The resulting QD solution was dialyzed against Milli-Q water for 4 h then concentrated to 15 mL using a rotary evaporator. QDs were precipitated using MeOH/CHCl_3 _(1:1, v/v) then collected by centrifugation. The QDs were washed with MeOH/CHCl_3 _(1:1, v/v) two times then dried under vacuum. The QDs were used as solutions either in deionized water or in PBS buffer.

### Preparation of NAC capped (-) CdTe

Cadmium perchlorate hydrate (500 μL, 1 M aqueous solution) and N-acetylcysteine (400 mg, 2.45 mmol) were dissolved in 200 mL of N_2 _saturated Milli-Q water. The pH of the solution was adjusted to 10.5 with 1N NaOH aqueous solution prior to addition of an aliquot of the previously prepared NaHTe solution (200 μL). The reaction mixture was heated to reflux for 25 min under N_2_. The resulting QD solution was dialyzed against Milli-Q water for 4 h then processed as above.

### Conjugation of NAC to cysteamine capped QD

Solutions of NAC (4 mM) were freshly prepared in water mixed with cysteamine capped (+) CdTe QD in water (2 mg/mL, λem = 542 nm) followed by 1-ethyl-3-(3'-dimethylaminopropyl)carbodiimide (EDC; 12 mg, 77.3 μmol) addition. The reaction mixture was incubated for 3 h at room temperature with occasional shaking. The mixture was purified by dialysis against water for 4 h. The emission wavelength of the resulting solution was 533 nm. The NAC-conjugated QDs were used as a solution in water. Zeta potentials of all QD preparations were measured using Zetasizer Nano ZS (Malvern Instruments, Worchestershire, UK).

### Cell culture and treatments

The human neuroblastoma cell line SH-SY5Y was obtained from ATCC and cultured (37°C, 5% CO_2_) in DMEM medium containing phenol red and 10% FBS (Gibco, Burlington, ON, Canada). Cells were used at 2–8 passages. For spectrofluorometric and colorimetric assays, cells were cultured in 24-well plates (Sarstedt, Montreal, QC, Canada) at a density of 10^5 ^cells/cm^2^.

One hour prior to treatments, medium containing serum was aspirated, and cells washed with serum free medium. Fresh serum free medium was added to all wells, including the untreated control (Ctrl). An additional set of control cells, grown in 10% FBS, was used to account for changes in cell morphology, cell number and metabolic activity due to the serum withdrawal.

Cells were treated with QDs (5 μg/mL) for different time periods as specified in individual figure legends. QD solutions (5 μg/mL) were prepared from the stock (2 mg/mL) by dilution in serum free cell culture medium. Cells were incubated with QDs for a maximum of 24 h before biochemical analysis or live cell imaging.

NAC was dissolved in PBS (400 mM), and was added to the culture medium 2 h before QDs. All treatments were done in triplicates or quadruplicates in three or more independent experiments.

### Flow cytometry in determining cell viability, Fas expression and cellular uptake of QDs

SH-SY5Y cells were treated with 5 μg/ml QDs (NAC-modified and unmodified) and/or 2 mM NAC (as indicated) for 24 h at 37°C/5% CO_2 _in media supplemented with 10% FBS. Adherent and non-adherent cells were harvested and pooled so as not to lose apoptotic cells which may have detached from the plastic substrate. Cells were resuspended at 1 × 10^6 ^cells/ml in FACS buffer (PBS + 1 % FCS). Fas expression was determined by labeling cells with phycoerythrin (PE) conjugated anti-human Fas/CD95 (clone DX2, BD Biosciences), and PE conjugated isotype-matched control antibodies (mouse IgG1 kappa, BD Biosciences) for 30 min on ice. Cells were washed twice and resuspended in 300 μl FACS buffer. Samples analysed for viability and/or for quantum dot-associated fluorescence alone (FL1, PMT 488–540 nm) were not labeled with antibodies. 10,000 events per sample were acquired on a Becton Dickinson FACScan flow cytometer. Data were analyzed using CellQuest software. Fas expression was determined as follows: Net Fas expression = Fas mean fluorescence intensity (MFI) – isotype control MFI for each individual sample, then averages and standard deviations of three independent replicates were calculated.

### MTT assay

Colorimetric MTT (3-(4,5-dimethylthiazol-2-yl)-2,5-diphenyl tetrazolium bromide, Sigma) assays were performed to assess the mitochondrial activity of cells treated as described above. After 24 h treatment, media was removed and replaced with drug-free, serum-free media (500 μL/well). 50 μL of stock MTT (5 mg/mL) was added to each well and cells were then incubated for one hour at 37°C. Media were removed, cells were lysed and formazan dissolved with DMSO. Absorbance was measured at 595 nm using a Benchmark microplate reader (Bio-Rad, Mississauga, ON, Canada). All measurements were done in triplicates in three or more independent experiments.

### Lipid peroxidation

Cells were treated with the fluorescent dye BODIPY 581/591 C_11 _(BODIPY-C_11_, Molecular Probes), which inserts into lipid membranes and allows for quantitative assessment of oxidized versus non-oxidized lipids by fluorescing green or red, respectively. Cells were stained for 30 min with a 10 μM solution of BODIPY-C_11 _prior to QD treatment. After the QD treatment, lipids were extracted from the cells according to the Folch method [52] by incubating twice with a mixture of chloroform and methanol (2:1 (v/v)). After extraction, 0.2 volumes of 0.9% NaCl solution were added and the chloroform-containing phase was collected. After evaporating the chloroform and dissolving the lipids in isopropanol, spectrofluorometric readings were taken using the SpectraMax Gemini XS microplate spectrofluorometer (Molecular Devices Corporation, USA). Data were analyzed using the SOFTmax Pro 4.0 program. All values are presented as normalized means ± SEM relative to the respective serum-free control (taken as 100%).

### Confocal microscopy

Images were acquired with a Zeiss LSM 510 NLO inverted microscope. Cells were grown in 8-well chamber slides (Lab-Tek, Nalge Nunc International, Rochester, NY, USA). QDs were added to designated wells and the cells were incubated for the times indicated. Mitochondria were stained with MitoTracker Deep Red 633 (1 μM, 1 min, Molecular Probes; λ_ex _644 nm, λ_em _665 nm) and imaged using HeNe 633 nm excitation laser and LP 650 filter. Lipid peroxidation was visualized using BODIPY-C_11 _(Molecular Probes; non-oxidized: λ_ex _581 nm, λ_em _595 nm; oxidized: λ_ex _485 nm, λ_em _520 nm) with the Argon 488 nm excitation laser and LP 520 nm filter, and the HeNe 543 nm laser and LP 560 filter. Mitochondrial depolarization was determined using JC-1 (15 μM, 30 min, Molecular Probes; monomer: λ_ex _485 nm, λ_em _530 nm; aggregate: λ_ex _535 nm, λ_em _590 nm). The potential-sensitive color shift was monitored using the same set of lasers and filters as BODIPY-C_11_. Before imaging, cells were washed with PBS or with serum-free medium. No background fluorescence of cells was detected under the settings used. Images were acquired at a resolution of 512 × 512 and 1024 × 1024. Quadruplicate samples were analyzed in all the imaging experiments. Scan size was 146.2 μm × 146.2 μm. Figures were created using Adobe Photoshop.

### Statistical analysis

Data were analyzed using SYSTAT 10 (SPSS, Chicago, IL, USA). Statistical significance was determined by Student's t-tests with Bonferroni correction. Differences were considered significant where *p < 0.05, **p < 0.01, ***p < 0.001.

## Abbreviations

CdTe, cadmium telluride; NAC, N-acetylcysteine; QD, quantum dot; ROS, reactive oxygen species; FACS, fluorescence-activated cell sorting; MTT, 3-(4,5-dimethylthiazol-2-yl)-2,5-diphenyltetrazolium bromide; BODIPY-C_11_, 4,4-difluoro-5-(4-phenyl-1,3-butadienyl)-4-bora-3a,4a-diaza-*s*-indacene-3-undecanoic acid; JC-1, 5,5',6,6'-tetrachloro-1,1',3,3'-tetraethylbenzimidazolylcarbocyanine iodide;

## Competing interests

The author(s) declare that they have no competing interests.

## Authors' contributions

DM initiated and guided these studies. DM drafted and AOC finalized the manuscript. AOC, SJC, JD and JL carried out the experiments. All authors read and approved the final manuscript.

## Supplementary Material

Additional file 1 PL spectra (stability) of CdTe nanoparticles in water and PBS. Click here for file

## References

[B1] Schwab M, Westermann F, Hero B, Berthold F (2003). Neuroblastoma: biology and molecular and chromosomal pathology. Lancet Oncol.

[B2] Brodeur GM (2003). Neuroblastoma: biological insights into a clinical enigma. Nat Rev Cancer.

[B3] Henry MC, Tashjian DB, Breuer CK (2005). Neuroblastoma update. Curr Opin Oncol.

[B4] Kushner BH (2004). Neuroblastoma: a disease requiring a multitude of imaging studies. J Nucl Med.

[B5] Nishiyama N, Kataoka K (2006). Current state, achievements, and future prospects of polymeric micelles as nanocarriers for drug and gene delivery. Pharmacol Ther.

[B6] Vicent MJ, Duncan R (2006). Polymer conjugates: nanosized medicines for treating cancer. Trends in biotechnology.

[B7] Portney NG, Ozkan M (2006). Nano-oncology: drug delivery, imaging, and sensing. Anal Bioanal Chem.

[B8] Leary SP, Liu CY, Apuzzo ML (2006). Toward the emergence of nanoneurosurgery: part II--nanomedicine: diagnostics and imaging at the nanoscale level. Neurosurgery.

[B9] Giepmans BN, Adams SR, Ellisman MH, Tsien RY (2006). The fluorescent toolbox for assessing protein location and function. Science.

[B10] Pinaud F, Michalet X, Bentolila LA, Tsay JM, Doose S, Li JJ, Iyer G, Weiss S (2006). Advances in fluorescence imaging with quantum dot bio-probes. Biomaterials.

[B11] Gao X, Cui Y, Levenson RM, Chung LW, Nie S (2004). In vivo cancer targeting and imaging with semiconductor quantum dots. Nat Biotechnol.

[B12] Lovric J, Cho SJ, Winnik FM, Maysinger D (2005). Unmodified cadmium telluride quantum dots induce reactive oxygen species formation leading to multiple organelle damage and cell death. Chem Biol.

[B13] Hardman R (2006). A toxicologic review of quantum dots: toxicity depends on physicochemical and environmental factors. Environ Health Perspect.

[B14] Lovric J, Bazzi HS, Cuie Y, Fortin GRA, Winnik FM, Maysinger D (2005). Differences in subcellular distribution and toxicity of green and red emitting CdTe quantum dots. Journal of Molecular Medicine-Jmm.

[B15] Ryman-Rasmussen JP, Riviere JE, Monteiro-Riviere NA (2007). Surface coatings determine cytotoxicity and irritation potential of quantum dot nanoparticles in epidermal keratinocytes. J Invest Dermatol.

[B16] Vogt M, Bauer MK, Ferrari D, Schulze-Osthoff K (1998). Oxidative stress and hypoxia/reoxygenation trigger CD95 (APO-1/Fas) ligand expression in microglial cells. FEBS Lett.

[B17] Golstein P, Kroemer G (2007). Cell death by necrosis: towards a molecular definition. Trends Biochem Sci.

[B18] Nagata S, Golstein P (1995). The Fas Death Factor. Science.

[B19] Raoul C, Pettmann B, Henderson CE (2000). Active killing of neurons during development and following stress: a role for p75(NTR) and Fas?. Curr Opin Neurobiol.

[B20] Martin-Villalba A, Herr I, Jeremias I, Hahne M, Brandt R, Vogel J, Schenkel J, Herdegen T, Debatin KM (1999). CD95 ligand (Fas-L/APO-1L) and tumor necrosis factor-related apoptosis-inducing ligand mediate ischemia-induced apoptosis in neurons. J Neurosci.

[B21] Ju ST, Panka DJ, Cui H, Ettinger R, el-Khatib M, Sherr DH, Stanger BZ, Marshak-Rothstein A (1995). Fas(CD95)/FasL interactions required for programmed cell death after T-cell activation. Nature.

[B22] Thorburn A (2004). Death receptor-induced cell killing. Cell Signal.

[B23] Wallach D, Varfolomeev EE, Malinin NL, Goltsev YV, Kovalenko AV, Boldin MP (1999). Tumor necrosis factor receptor and Fas signaling mechanisms. Annu Rev Immunol.

[B24] Moreira PI, Smith MA, Zhu X, Nunomura A, Castellani RJ, Perry G (2005). Oxidative stress and neurodegeneration. Ann N Y Acad Sci.

[B25] Foster KA, Galeffi F, Gerich FJ, Turner DA, Muller M (2006). Optical and pharmacological tools to investigate the role of mitochondria during oxidative stress and neurodegeneration. Prog Neurobiol.

[B26] Zafarullah M, Li WQ, Sylvester J, Ahmad M (2003). Molecular mechanisms of N-acetylcysteine actions. Cell Mol Life Sci.

[B27] Cho SJ, Maysinger D, Jain M, Roder B, Hackbarth S, Winnik FM (2007). Long-term exposure to CdTe quantum dots causes functional impairments in live cells. Langmuir.

[B28] Lopez E, Arce C, Oset-Gasque MJ, Canadas S, Gonzalez MP (2006). Cadmium induces reactive oxygen species generation and lipid peroxidation in cortical neurons in culture. Free radical biology & medicine.

[B29] Derfus AM, Chan WCW, Bhatia SN (2004). Probing the cytotoxicity of semiconductor quantum dots. Nano Letters.

[B30] Michalet X, Pinaud FF, Bentolila LA, Tsay JM, Doose S, Li JJ, Sundaresan G, Wu AM, Gambhir SS, Weiss S (2005). Quantum dots for live cells, in vivo imaging, and diagnostics. Science.

[B31] Zhelev Z, Ohba H, Bakalova R (2006). Single quantum dot-micelles coated with silica shell as potentially non-cytotoxic fluorescent cell tracers. J Am Chem Soc.

[B32] Clarke SJ, Hollmann CA, Zhang Z, Suffern D, Bradforth SE, Dimitrijevic NM, Minarik WG, Nadeau JL (2006). Photophysics of dopamine-modified quantum dots and effects on biological systems. Nat Mater.

[B33] Samia AC, Dayal S, Burda C (2006). Quantum dot-based energy transfer: perspectives and potential for applications in photodynamic therapy. Photochemistry and photobiology.

[B34] Voura EB, Jaiswal JK, Mattoussi H, Simon SM (2004). Tracking metastatic tumor cell extravasation with quantum dot nanocrystals and fluorescence emission-scanning microscopy. Nat Med.

[B35] Kirchner C, Liedl T, Kudera S, Pellegrino T, Munoz Javier A, Gaub HE, Stolzle S, Fertig N, Parak WJ (2005). Cytotoxicity of colloidal CdSe and CdSe/ZnS nanoparticles. Nano Lett.

[B36] Hoshino A, Fujioka K, Oku T, Suga M, Sasaki YF, Ohta T, Yasuhara M, Suzuki K, Yamamoto K (2004). Physicochemical Properties and Cellular Toxicity of Nanocrystal Quantum Dots Depend on Their Surface Modification. Nano Lett.

[B37] Sheng W, Kim S, Lee J, Kim SW, Jensen K, Bawendi MG (2006). In-situ encapsulation of quantum dots into polymer microspheres. Langmuir.

[B38] van Vlerken LE, Amiji MM (2006). Multi-functional polymeric nanoparticles for tumour-targeted drug delivery. Expert Opin Drug Deliv.

[B39] Iverson SL, Orrenius S (2004). The cardiolipin-cytochrome c interaction and the mitochondrial regulation of apoptosis. Arch Biochem Biophys.

[B40] Sen T, Sen N, Tripathi G, Chatterjee U, Chakrabarti S (2006). Lipid peroxidation associated cardiolipin loss and membrane depolarization in rat brain mitochondria. Neurochem Int.

[B41] Kroemer G, Galluzzi L, Brenner C (2007). Mitochondrial membrane permeabilization in cell death. Physiological reviews.

[B42] Shibata T, Iio K, Kawai Y, Shibata N, Kawaguchi M, Toi S, Kobayashi M, Kobayashi M, Yamamoto K, Uchida K (2006). Identification of a lipid peroxidation product as a potential trigger of the p53 pathway. J Biol Chem.

[B43] Beyaert R, Van Loo G, Heyninck K, Vandenabeele P (2002). Signaling to gene activation and cell death by tumor necrosis factor receptors and Fas. Int Rev Cytol.

[B44] Curtin JF, Cotter TG (2003). Live and let die: regulatory mechanisms in Fas-mediated apoptosis. Cell Signal.

[B45] Peter ME, Krammer PH (2003). The CD95(APO-1/Fas) DISC and beyond. Cell Death Differ.

[B46] Matsushita K, Wu Y, Qiu J, Lang-Lazdunski L, Hirt L, Waeber C, Hyman BT, Yuan J, Moskowitz MA (2000). Fas receptor and neuronal cell death after spinal cord ischemia. J Neurosci.

[B47] Martin LJ, Chen K, Liu Z (2005). Adult motor neuron apoptosis is mediated by nitric oxide and Fas death receptor linked by DNA damage and p53 activation. J Neurosci.

[B48] Raoul C, Estevez AG, Nishimune H, Cleveland DW, deLapeyriere O, Henderson CE, Haase G, Pettmann B (2002). Motoneuron death triggered by a specific pathway downstream of Fas. potentiation by ALS-linked SOD1 mutations. Neuron.

[B49] Yang JY, Xia W, Hu MC (2006). Ionizing radiation activates expression of FOXO3a, Fas ligand, and Bim, and induces cell apoptosis. Int J Oncol.

[B50] Maysinger D, Lovric J, Eisenberg A, Savic R (2006). Fate of micelles and quantum dots in cells. Eur J Pharm Biopharm.

[B51] Fischer HC, Liu L, Pang KS, Chan WC (2006). Pharmacokinetics of nanoscale quantum dots: in vivo distribution, sequestration, and clearance in the rat. Adv Funct Mater.

[B52] Folch J, Lees M, Sloane Stanley GH (1957). A simple method for the isolation and purification of total lipides from animal tissues. J Biol Chem.

