# Supplementary material for: Quantum dot-induced cell death involves Fas upregulation and lipid peroxidation in human neuroblastoma cells
Source: J Nanobiotechnology. 2007 Feb 12;5:1. doi: 10.1186/1477-3155-5-1 (PMC1802956; doi:10.1186/1477-3155-5-1)
Supplement: Additional file 1 — PL spectra (stability) of CdTe nanoparticles in water and PBS. [file 1477-3155-5-1-S1.ppt]

## Slide 1
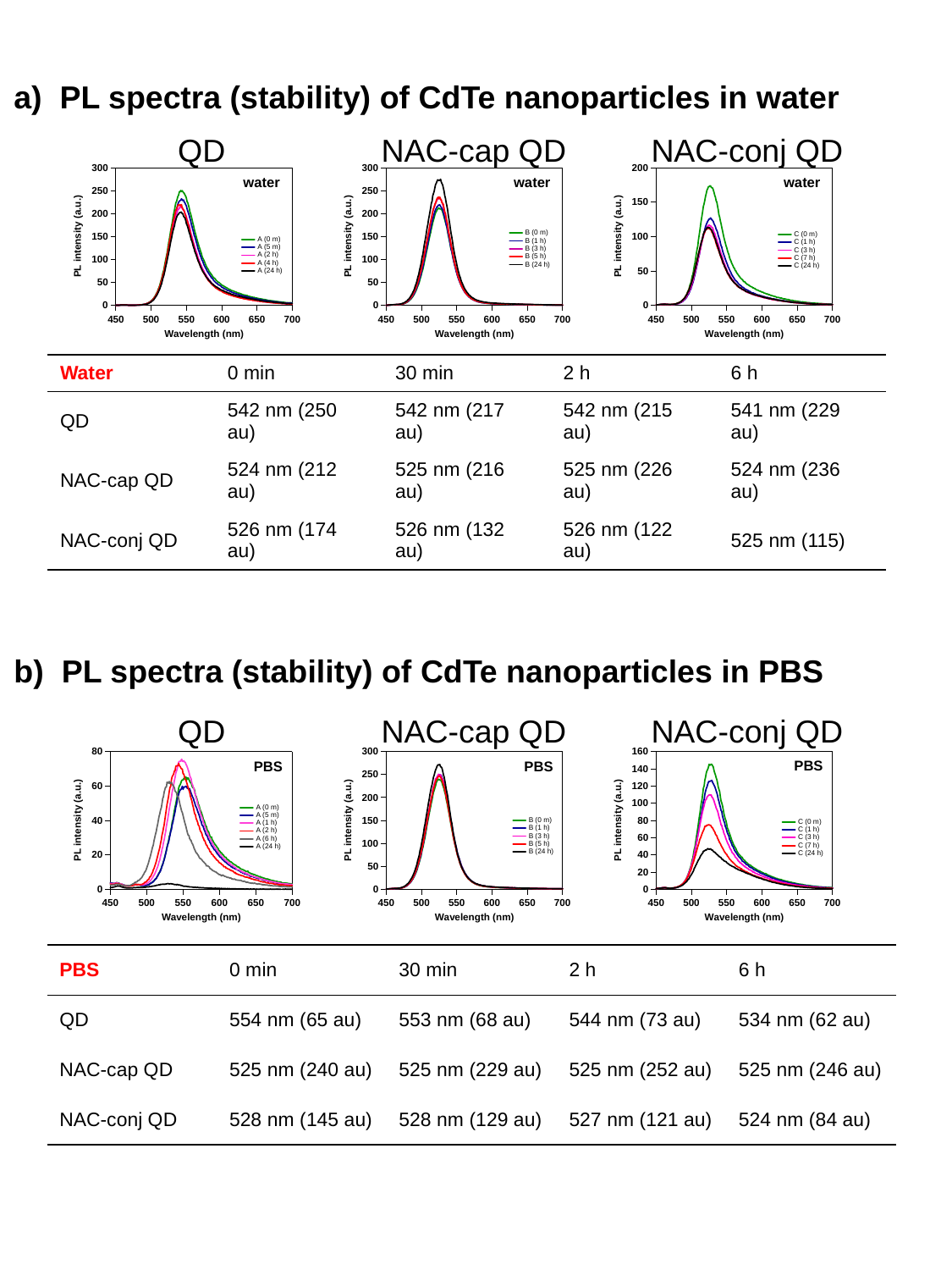

a) PL spectra (stability) of CdTe nanoparticles in water
QD
NAC-cap QD
NAC-conj QD
water
water
water
| Water | 0 min | 30 min | 2 h | 6 h |
| --- | --- | --- | --- | --- |
| QD | 542 nm (250 au) | 542 nm (217 au) | 542 nm (215 au) | 541 nm (229 au) |
| NAC-cap QD | 524 nm (212 au) | 525 nm (216 au) | 525 nm (226 au) | 524 nm (236 au) |
| NAC-conj QD | 526 nm (174 au) | 526 nm (132 au) | 526 nm (122 au) | 525 nm (115) |
b) PL spectra (stability) of CdTe nanoparticles in PBS
QD
NAC-cap QD
NAC-conj QD
PBS
PBS
PBS
| PBS | 0 min | 30 min | 2 h | 6 h |
| --- | --- | --- | --- | --- |
| QD | 554 nm (65 au) | 553 nm (68 au) | 544 nm (73 au) | 534 nm (62 au) |
| NAC-cap QD | 525 nm (240 au) | 525 nm (229 au) | 525 nm (252 au) | 525 nm (246 au) |
| NAC-conj QD | 528 nm (145 au) | 528 nm (129 au) | 527 nm (121 au) | 524 nm (84 au) |
